# Whole Transcriptome Analysis of Mesenchyme Tissue in Sika Deer Antler Revealed the CeRNAs Regulatory Network Associated With Antler Development

**DOI:** 10.3389/fgene.2019.01403

**Published:** 2020-02-18

**Authors:** Ruobing Han, Lei Han, Shengnan Wang, Heping Li

**Affiliations:** College of Wildlife and Protected Area, Northeast Forestry University, Harbin, China

**Keywords:** antler, development, DE mRNAs, DE miRNAs, DE lncRNAs, ceRNET

## Abstract

Deer antler is the only completely regenerable organ in mammals. During the rapid growth period, the antler proliferates even faster than cancerous tissue growth. However, the proliferation and development of antler have been in a stable and controllable growth cycle. In this study, we analyzed the time series expression data of nine samples from mesenchyme layer in three male sika deer in the early period of the antler with a saddle-like appearance (30 days), the rapid growth period of the antler with two branches (60 days), and the final period of the antler with three branches (90 days). Whole Transcriptome sequencing results show that in the 30 d versus 60 d group, 1,464 genes, 85 long noncoding RNAs (lncRNAs), and 61 miRNAs were identified as differentially expressed; 1,748 genes, 138 lncRNAs, and 78 miRNAs were identified as differentially expressed in 30d versus 90d group; and 816 differentially expressed genes (DEGs), 49 differentially expressed lncRNAs (DE lncRNAs), and 24 differentially expressed miRNA (DE miRNAs) were identified in 60d versus 90d group. A total of 182 miRNA-mRNA interaction pairs and 89 miRNA-lncRNA interaction pairs were screened from DEGs, DE miRNAs, and DE lncRNAs to construct the ceRNA regulatory network (ceRNET). In summary, we identified candidate mRNAs, miRNAs and lncRNAs that regulate the development of antler tip. It may lay the foundation for further investigating the molecular mechanism of antler rapid growth and development.

## Introduction

Antlers are male secondary sexual characteristics (except in reindeer). It is the unique appendages organ that can undergo periodic regeneration and shedding in mammalians. The annual cycle of antler growth starts in spring, and then antler begins to elongate rapidly and forms lateral branches. During this time, the growth rate of the sika deer antlers can reach 12.5 mm/d (Li et al., 2014). At the end of the summer, the antler grows out of the second lateral branch and then stops growing, laying the foundation for the complete calcification of the antlers in the upcoming breeding season.

Rapid development of antler is a strictly regulated process, which requires precise control of gene expression by regulatory factors. MicroRNA (miRNA), a class of non-coding RNAs, is small RNAs (18–24 nucleotide) that are thought to regulate gene expression through sequence-specific base pairing with target mRNAs (Bartel, 2004; Bartel, 2009). Chen et al. identified 399 miRNAs by deep sequencing of red deer cartilage tissue, 54 miRNA of which were unique to red deer (Chen et al., 2015). Ba et al. constructed miRNA expression profiles of pedicle periosteum stem cells in the potentiated and dormant periods by next generation sequencing technology (Ba et al., 2016). Some studies reveal that the miRNAs, such as miR-1, miR-18a, let-7a, and let-7f, positively regulate proliferation of the cell residents in the growth center of growing antler in the sika deer (Hu et al., 2013; Hu et al., 2014a; Hu et al., 2014b). Thus, miRNAs play crucial roles during the antler development. However, the fundamental mechanism of antler's rapid development is still poorly understood.

(Long non-coding RNA) lncRNA has a wide range of regulatory roles in gene expression, reproduction, cell growth and development, as well as the development of multiple cancers (Carrington and Ambros, 2003; Williams, 2008; Pandey et al., 2009; Ma et al., 2010; Yong et al., 2011; Ming et al., 2012; Clark et al., 2014). An increasingly evidence has showed that lncRNA regulate gene expression by acting as a competing endogenous RNA (ceRNA). To our knowledge, there are no reports of the involvement of lncRNAs in antler development. Therefore, the study of lncRNA during the antler development through RNA-seq technology can help to reveal the mechanism of antler fast development from the molecular perspective. CeRNAs are RNAs that can actively crosstalk with each other by microRNA recognition elements (MREs) to regulate their respective expression levels, MREs can be viewed as the letter of an “RNA language” among them (Xu et al., 2016). CeRNA hypothesis can help us better explain the intricate interplay among diverse RNAs (mRNA, miRNA, lncRNA, and circRNA) (Xu et al., 2016).

The ceRNET has been shown in the regulation of multiple signaling pathways, including MAPK, HIF-1, RAS, and PI3K/AKT, etc (Tian and Xiao, 2013), which is involved in biological processes such as cell proliferation, apoptosis, cell cycle, invasion, and metastasis. Protein-coding RNAs and lncRNAs can act as ceRNAs to communicate with each other by competitively binding to miRNAs sites according to ceRNA hypothesis. Currently, ceRNET is mainly used to solve human cancer treatment, among which the typical representative is the construction of PTEN's ceRNET. In addition, ceRNET has also been applied to explore biological characteristics in animal and plant research (Qiu et al., 2018; Bai et al., 2019; He et al., 2019; Li et al., 2019; Wang et al., 2019a). It is reasonable to hypothesized that ceRNET would be an exactly effective methods to reflect the complete and accurate regulate process of the antler rapidly development.

Antlers develop as extensions from the pedicles, which are paired permanent outgrowths of the frontal bones (Landete-Castillejos et al., 2019). The previous study has showed that the tip tissue of antler, especially the mesenchyme cell, is the growth center of antler (Banks and Newbrey, 1982). In this study, in order to explore the mechanism of antler growth and development, the mesenchymal tissues of antler from three periods of three male deer was collected for whole transcriptome sequencing. We identified DEGs, DE miRNAs, and DE lncRNAs and then constructed ceRNET for understanding the complexity of ceRNA crosstalk and competition in these three periods of antler development. This is the first investigation to create a profile for the ceRNET of antler whole transcriptome. Interpretation of interactions between different RNAs would provide a significant insight into rapid development of antler and a basis for revealing the regulation mechanism of rapid proliferation of antler cells in rapid growth period. Furthermore, Elucidating the molecular mechanisms of rapid development in antler may provide a solid reference for the future studies on human cancer treatment (Wang et al., 2019b), and maybe even for limb regeneration.

## Materials and Methods

### Sample Collection

The samples were purchased from a commercial farm in Harbin, China. Mesenchymal tissues in sika deer of three periods of three healthy adult males deer aged 5 years were collected for RNA isolation, which were placed into clean RNase-free Eppendorf tubes and immediately stored in liquid nitrogen. When the antler grew to a saddle-like appearance, the mesenchymal tissues in left antler were collected as early period (30 d) (Takeda-Okuda et al., 2019). The 60 d is the mesenchymal tissues in right antler, which grew with two branches. When the antler grew with three-branched, the mesenchymal tissues in left antler were collected as the 90 d. All experimental designs and animal handling were approved by the Institutional Animal Care and Use Committee of Northeast Forestry University (UT-31; 90 20 June 2014).

### RNA Extraction and Quality Control

RNA was extracted from nine samples using TRIzol reagent (TaKaRa, Dalian, China) according to the manufacturer's instructions. The concentration and purity of the RNA were evaluated with NanoDrop 2000 spectrophotometer, gel electrophoresis, and RNA integrity (RIN). The RIN of nine samples ranged from 8.6 to 9.3 (Marcus et al., 2006), indicating that the RNAs were intact and could be used in subsequent experiments and bioinformatics analysis.

### Libraries Construction and Sequencing

LncRNA libraries and small RNA libraries were construction, respectively. LncRNA libraries were construction then sequenced using Illumina HiSeqTM 4000. In order to obtain the high quality clean reads for subsequent analysis, we performed rigorous screening on the results obtained from lncRNA sequencing. Clean reads after filtration were matched to the reference genome of *Cervus elaphus* using software Tophat2 (v2.1.1) (Trapnell et al., 2009).

Small RNA libraries were constructed and then sequenced using Illumina HiSeqTM 2500. Meanwhile, clean tags were obtained mainly by removing reads containing more than one low quality (Q-value ≤ 20) base or containing unknown nucleotides(N), 3' and 5' adapters but no small RNA fragment between them, and reads shorter than 18nt (not include adapters), etc.

### Identification of DEGs, DE miRNAs, and DE lncRNAs

Samples from three male sika deer were used as biological replicates to ensure the accuracy and reliability of RNA-seq result. FPKM (Mortazavi et al., 2008) was used to quantify the lncRNAs or protein-coding RNAs expression levels, and TPM (Zhou et al., 2010) was used to quantify the miRNAs expression levels. Each of the three periods was compared to the others and DEGs, DE miRNAs and DE lncRNAs were identified. The threshold as p value < 0.05 and absolute fold-change values > 1 in any of the pairwise comparisons were considered significantly differentially expressed.

### Target Gene Prediction and Enrichment Analysis

RNAhybrid (v2.1.2) + svm_-_light (v6.01), Miranda (v3.3a), and TargetScan (Version: 7.0) were used to predict the target genes of miRNA. The intersection of the predicted results obtained by three methods was considered as the final predicted results of target genes. MiRNA-mRNA interaction pairs and miRNA-lncRNA interaction pairs were obtained from the results of miRNA target gene prediction.

**Figure 1:**
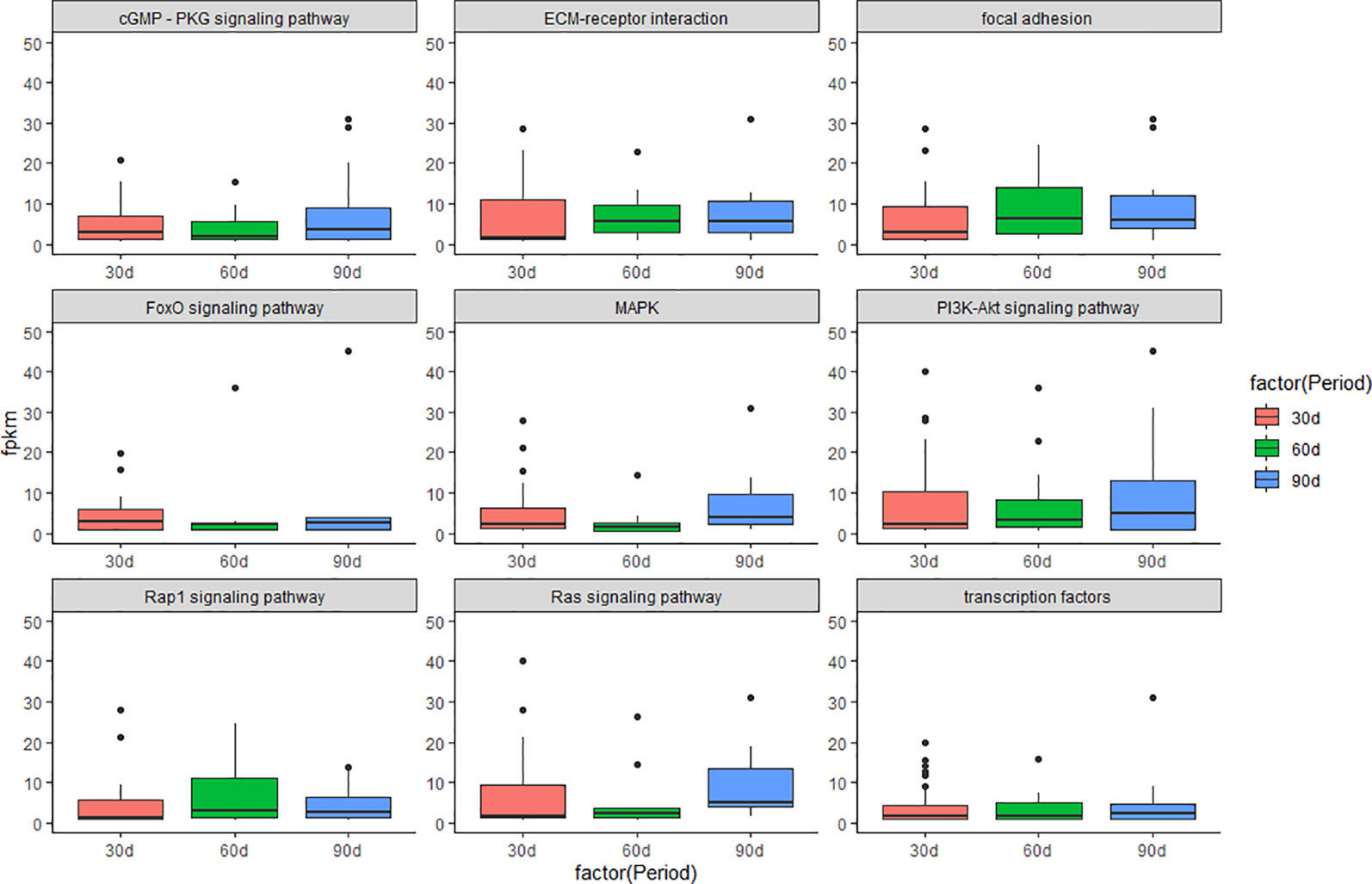
Boxplot of signaling pathway in the three periods during the antler development. The *y*-axis shows the FPKM of transcript.

Gene Ontology (GO: molecular function, cellular component, and biological process) enrichment analyses were performed on DEGs in three comparison groups with software DIVID (Ashburner et al., 2000; Kanehisa et al., 2016; Zhan et al., 2016). KEGG (Kyoto Encyclopedia of Genes and Genomes) enrichment analyses were performed on DEGs in three comparison groups using a hypergeometric test (Han et al., 2017). In addition, KEGG enrichment analyses were performed on the DEGs that enriched in the GO term of “development”. The *p* values were obtained through hypergeometric analyses corrected by the *q* values. Both GO terms and KEGG pathways were considered to be significantly enriched with *q* < 0.05. We further investigated the molecular mechanism of antler development by studying the changes in the expression levels of several important signaling pathways during the three periods of antler development. The visualization results are presented in boxplot (**Figure 1**).

### Construction of ceRNET

CeRNET was constructed to understand the crosstalk between protein-coding RNAs and non-coding RNAs in the process of antler development. DEGs enriched in the GO term of “development” were screened to construct the ceRNET. Among them, Pearson correlation coefficient (PCC) was evaluated for expression correlation between miRNA and target gene. Pairs with PCC < -0.7 and p < 0.05 were selected as co-expressed negatively miRNA-mRNA interaction pairs. Combined with results of miRNA-mRNA interaction pairs and miRNA-lncRNA interaction pairs. MiRNA-mRNA negative interaction pairs correlation with the antler development were screened in each comparison group, and then miRNA-lncRNA interaction pairs sharing the same MRE elements were screened to construct the co-expression regulatory network, which had correlation with the antler development. The visualization results were presented by software Cytoscape (v3.3.0) (Shannon et al., 2003).

### Validation by Real Time Quantitative PCR (qRT-PCR)

To verify the reliability of RNA-seq results, a total of 6 DEGs, 6 DE miRNAs, and 6 DE lncRNAs were randomly selected to validate using qRT-PCR in the three comparison groups (30 d versus 60 d, 30 d versus 90 d, and 60 d versus 90 d). The primer sequences were designed with NCBI primer blast (**Supplementary Table S1**). The miRNA primer sequence was designed according to the Mir-X ™ miRNA First Strand Synthesis and SYBR^®^ qRT-PCR User Manual (Takara) and the mRQ 3 ‘Primer from the kit was used as the reverse primer. QRT-PCR were carried out based on TB Green™ Premix Ex Taq™ (Tli RNaseH Plus). GAPDH (Livak and Schmittgen, 2001) and U6 snRNA as the housekeeping gene to validate the DEGs, DE lncRNAs, and DE miRNAs.

The PCR reaction mixture contained 10 μl TB Green Premix Ex TaqII (2×), 0.8 μl PCR Forward Primer (10μM), 0.8 μl PCR Reverse Primer (10 μM), 0.4 μl ROX Reference Dye (50×), 2 μl cDNA, and ddH_2_O up to a total volume of 20 μl. The qRT-PCR amplification was performed by pre-denaturation at 95°C for 30 s, followed by 30 cycles of 95°C for 3 s and 60°C for 30 s. Relative expressions were calculated using the 2^-ΔΔct^ method (Arocho et al., 2006). The results of qRT-PCR were consistent with the results of RNA-seq, which proved the reliability of sequencing results and vice versa (**Supplementary Figure S1**).

## Result

### Overview of RNA Sequencing

RNA-seq and small RNA-seq were performed on a total of nine samples from three periods of three male sika deer. RNA-seq results were filtered to yield 99.12% high quality clean reads for subsequent bioinformatics analysis. A total of 123 Gb of clean reads was obtained. More than 96.25% of the data yielded a high-quality score (Q30). The mapping ratio on the *Cervus elaphus* genome ranged from 71.17% to 73.03% (**Supplementary Table S2**).

The length distribution of clean tag was mainly between 21 bp and 23 bp. Tags in the nine samples were mapped and annotated, and the ratio of known miRNA was between 62.53% and 84.01%. Moreover, the mapping ratio of the tag sequence to the reference genome was between 67.77% and 77.57% (**Supplementary Table S3**).

### Identification of DEGs, DE miRNAs, and DE lncRNAs

**Figure 2:**
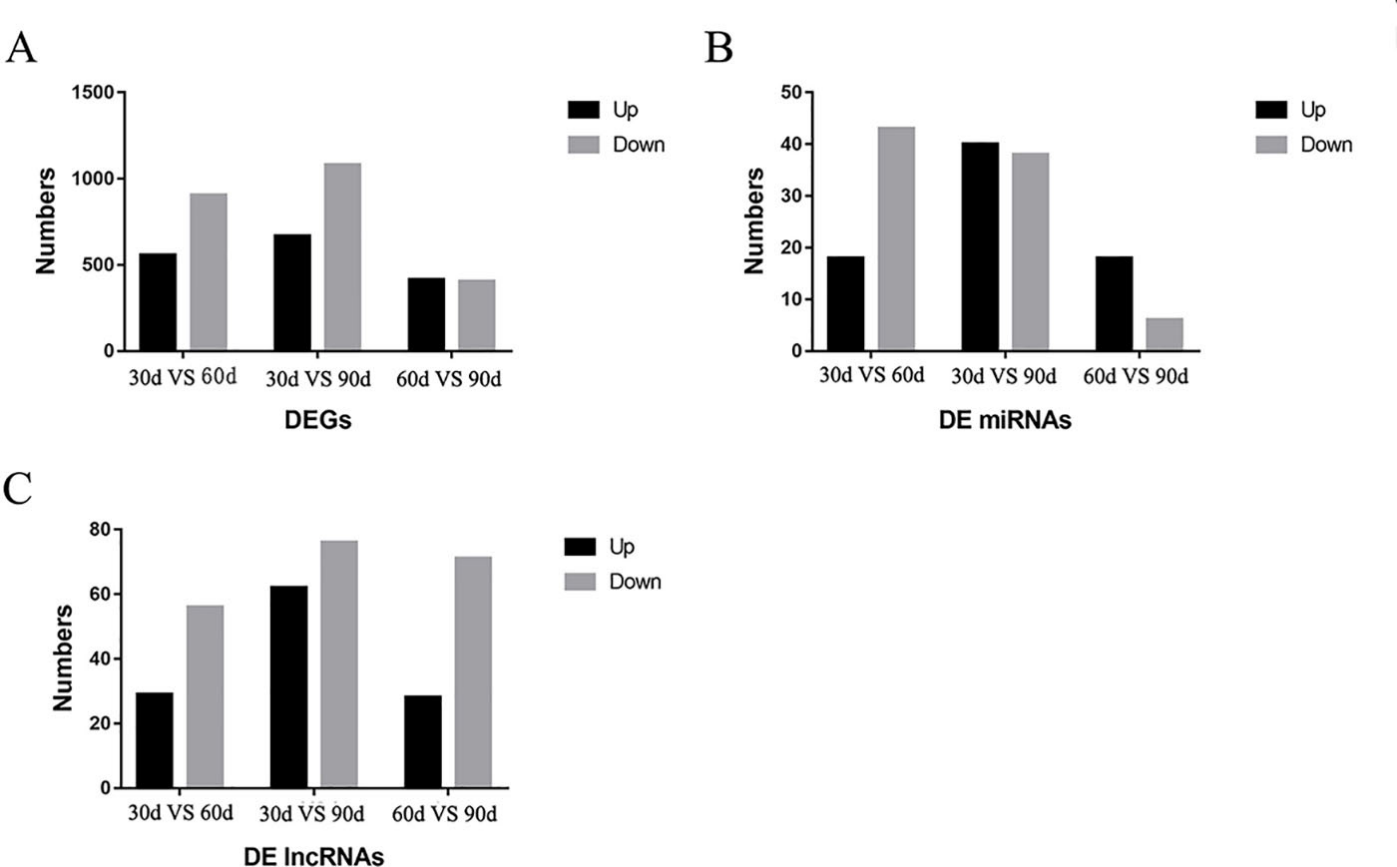
The results of differentially expressed genes (DEGs), differentially expressed miRNA (DE miRNAs), and differentially expressed lncRNAs (DE lncRNAs) in three comparison groups. **(A)** The DEGs were compared in the three comparison groups according to the up-regulation or down-regulation **(B)** The DE miRNAs were compared in the three comparison groups according to the up-regulation or down-regulation **(C)** The DE lncRNAs were compared in the three comparison groups according to the up-regulation or down-regulation.

A total of 35,925 mRNAs and 1,851 lncRNAs were identified from lncRNA libraries of nine samples, including 12,175 known and 23,750 novel mRNAs, and the 1,851 lncRNAs included 16 known lncRNAs, and 1,835 novel lncRNAs. All the 1,639 miRNAs were identified from small RNA libraries of nine samples, including 1,365 known and 274 novel miRNAs. Of the 1,365 known miRNAs, some miRNAs have been identified to be associated with antler development such as miR-296, miR-18, let-7, miR-145, miR-140, miR-133, miR-200, miR-196 (Ba et al., 2016), miR-21, miR-148, miR-99 (Chen et al., 2015), miR-205 and miR-203 (Hu et al., 2019). For the DEGs in the three comparison groups, the difference between 30 d and 90 d was the more significant, followed by 30 d versus 60 d. The DEGs, DE miRNAs and DE lncRNAs were compared between the three groups in pairs according to the up-regulation or down-regulation (**Figure 2**). It is worth noting that the number of down-regulated DEGs was higher than the number of up-regulated DEGs in 30 d versus 60 d and 30 d versus 90 d. The number of down-regulated DEGs was similar to that of up-regulated DEGs in 60 d versus 90 d.

### The Results of Target Gene Prediction

Target genes were predicted to explore the interaction between the mRNA and miRNA. The number of miRNA in nine SmallRNA libraries ranged from 819 to 970, the number of target genes between 6,265 and 6,333, a total of 1,639 different miRNAs were identified corresponding to 335,497 predicted miRNA-coding loci with the three methods (Mayer et al., 2014) with an average of 204 target genes per miRNA. The results claimed that one mRNA simultaneously targeted multiple miRNAs and vice versa (Bartel, 2004; Bartel, 2009), indicating the accuracy of the target gene prediction.

### The Results of Enrichment Analysis

**Figure 3:**
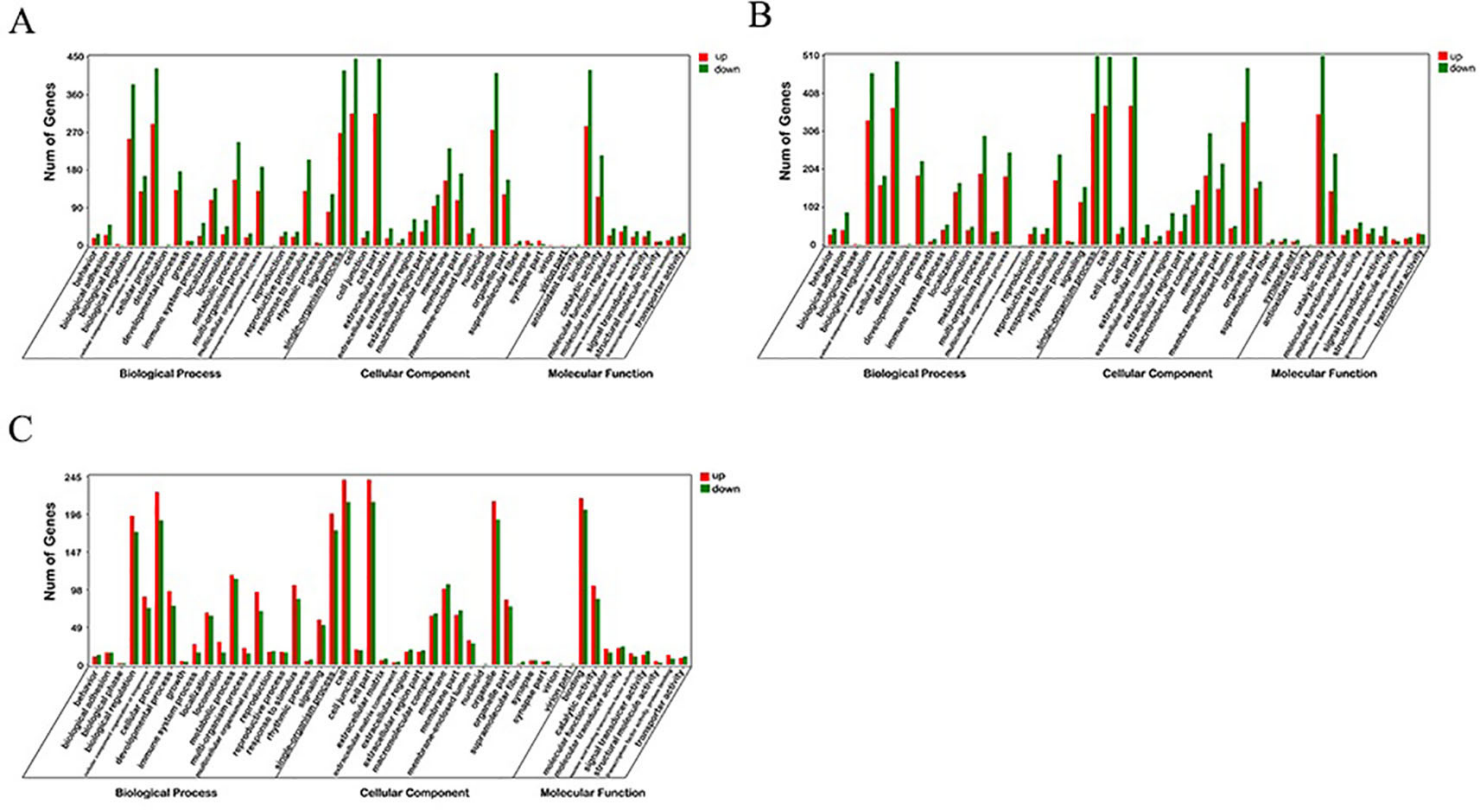
GO enrichment analysis of differentially expressed genes (DEGs). **(A)** 30 d versus 60 d; **(B)** 30 d versus 90 d; **(C)** 60 d versus 90 d.

GO enrichment analysis was carried out for the DEGs screened above, including three comparison groups (30 d versus 60 d, 30 d versus 90d, and 60d versus 90d). The results implied that the DEGs was mainly enriched in several GO terms, including “cellular process” (713 members), “cell” (761 members), “cell part” (761 members), and “binding” (704 members) terms in 30 d versus 60 d (**Figure 3**). In 30 d versus 90 d, “cell” (882 members), “cell part” (882 members), “cellular process” (864 members), and “single-organism process” (864 members) terms were predominant. In the 60 d versus 90 d, “cell” (453 members), “cell part” (453 members), “cellular process” (413 members), and “binding” (419 members) terms were predominant. We focused on the GO term of “development” in the process of antler development such as animal organ development, cellular developmental process, system development, and tissue development.

KEGG enrichment analyses were performed on the differential expressed transcripts, and it revealed that 30 d versus 60 d and 30 d versus 90 d were significantly enriched in Glycosaminoglycan binding proteins, cGMP/PKG signaling pathway, ECM-receptor interaction, some DEGs enriched in PI3K/AKT signaling pathway, and MAPK signaling pathway. For the 60 d versus 90 d, target genes were significantly enriched in AMPK signaling pathway, longevity regulating pathway, and PI3K/AKT signaling pathway. In addition, we also detected several interesting signaling pathways that may play a vital role in the antler rapid development, such as focal adhesion, mTOR signaling pathway, Hippo signaling pathway, and Wnt signaling pathway.

**Figure 4:**
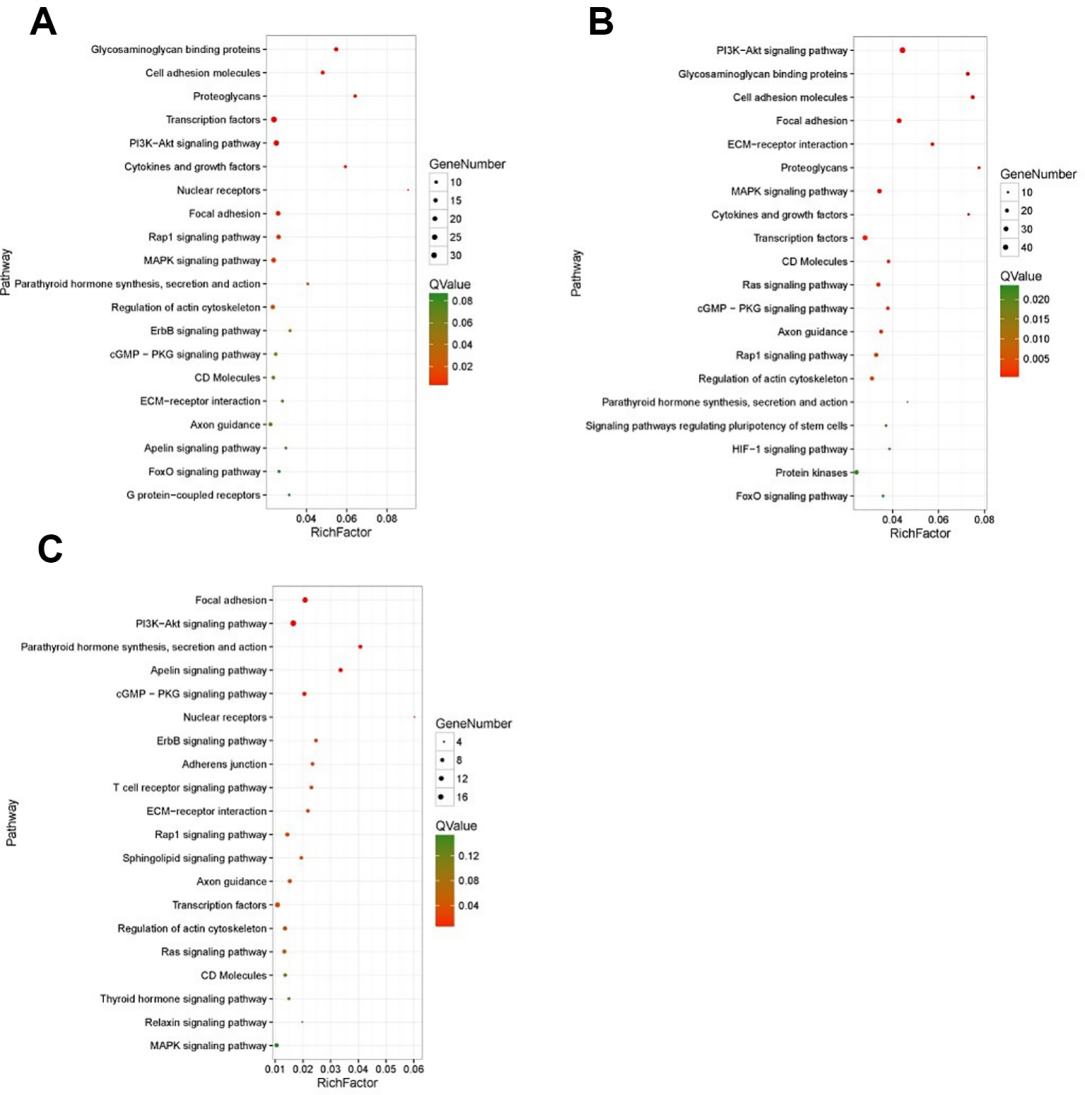
The results of top 20 KEGG enrichment in differentially expressed genes (DEGs) that enriched in GO term of “development”. **(A)** 30 d versus 60 d; **(B)** 30 d versus 90 d; **(C)** 60 d versus 90 d. The *x*-axis presents rich factor of DEGs in a category. The *y*-axis shows the specific pathway.

The results of KEGG on DEGs that were enriched in GO term of “development” showed that these DEGs were significantly enriched in the PI3K/AKT signaling pathway, cGMP/PKG signaling pathway, focal adhesion, and MAPK signaling pathway, etc. The results of top 20 KEGG enrichment are shown in **Figure 4**. cGMP/PKG signaling pathway, ECM-receptor interaction, focal adhesion, PI3K/AKT signaling pathway, Rap1 signaling pathway, and transcription factors may play important roles in the antler development, and the expression level of focal adhesion and Rap1 signaling pathway was higher in the 60 d. In contrast, the expression levels of cGMP/PKG signaling pathway, Fox0 signaling pathway, MAPK signaling pathway, PI3K/AKT signaling pathway, and Ras signaling pathway were higher in the 30 d and 90 d of antler development than 60 d.

### Construction of ceRNET

MiRNA-mRNA interaction pairs and miRNA-lncRNA interaction pairs related to the antler development were screened for constructing ceRNET. In details, 190, 455, and 39 co-expressed negatively miRNA-lncRNA interaction pairs were screened from 30 d versus 60 d, 30 d versus 90 d, and 60 d versus 90 d, respectively. At the same time, 1,683, 3,286, and 227 co-expressed negatively miRNA-mRNA interaction pairs were screened from 30 d versus 60 d, 30 d versus 90 d, and 60 d versus 90 d, respectively.

**Figure 5:**
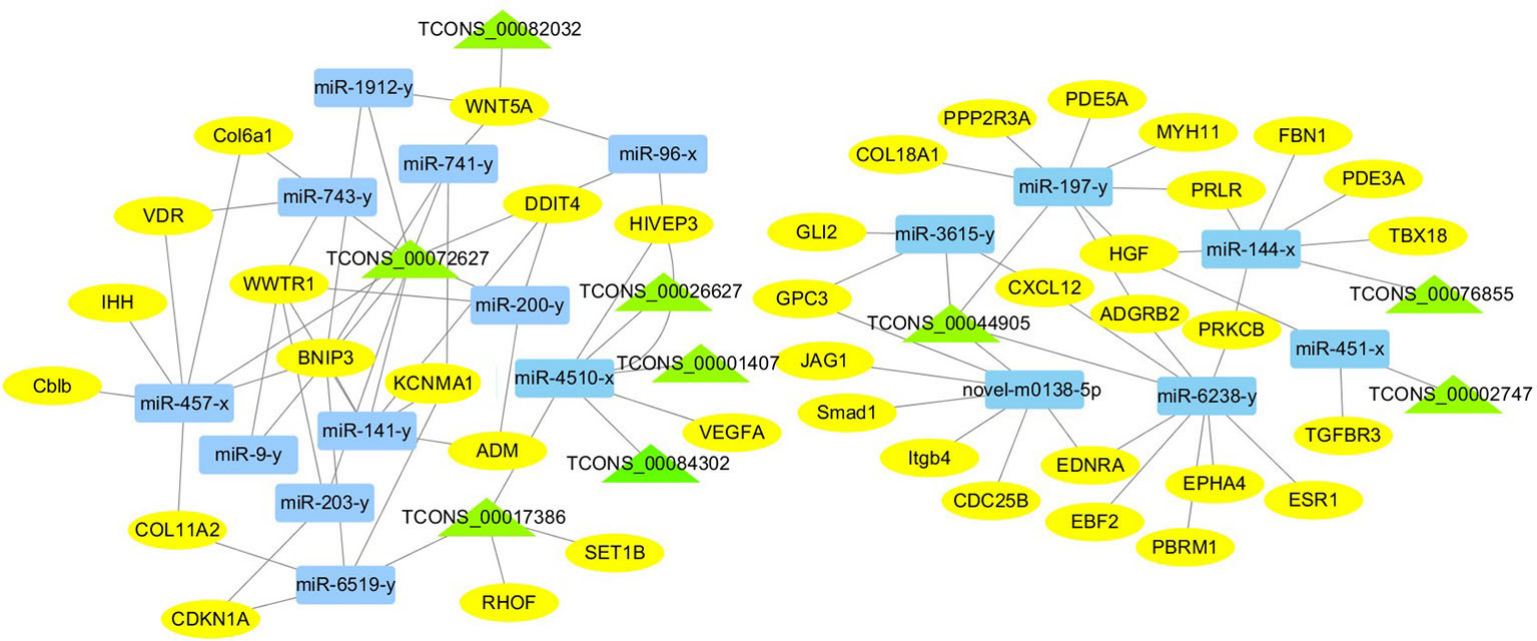
ceRNET of 30 d versus 60 d. Rectangles represent differentially expressed miRNAs, ellipses represent differentially expressed mRNAs, and the triangle represent differentially expressed lncRNAs. The gray lines represent the interactions between diverse RNAs.

**Figure 6:**
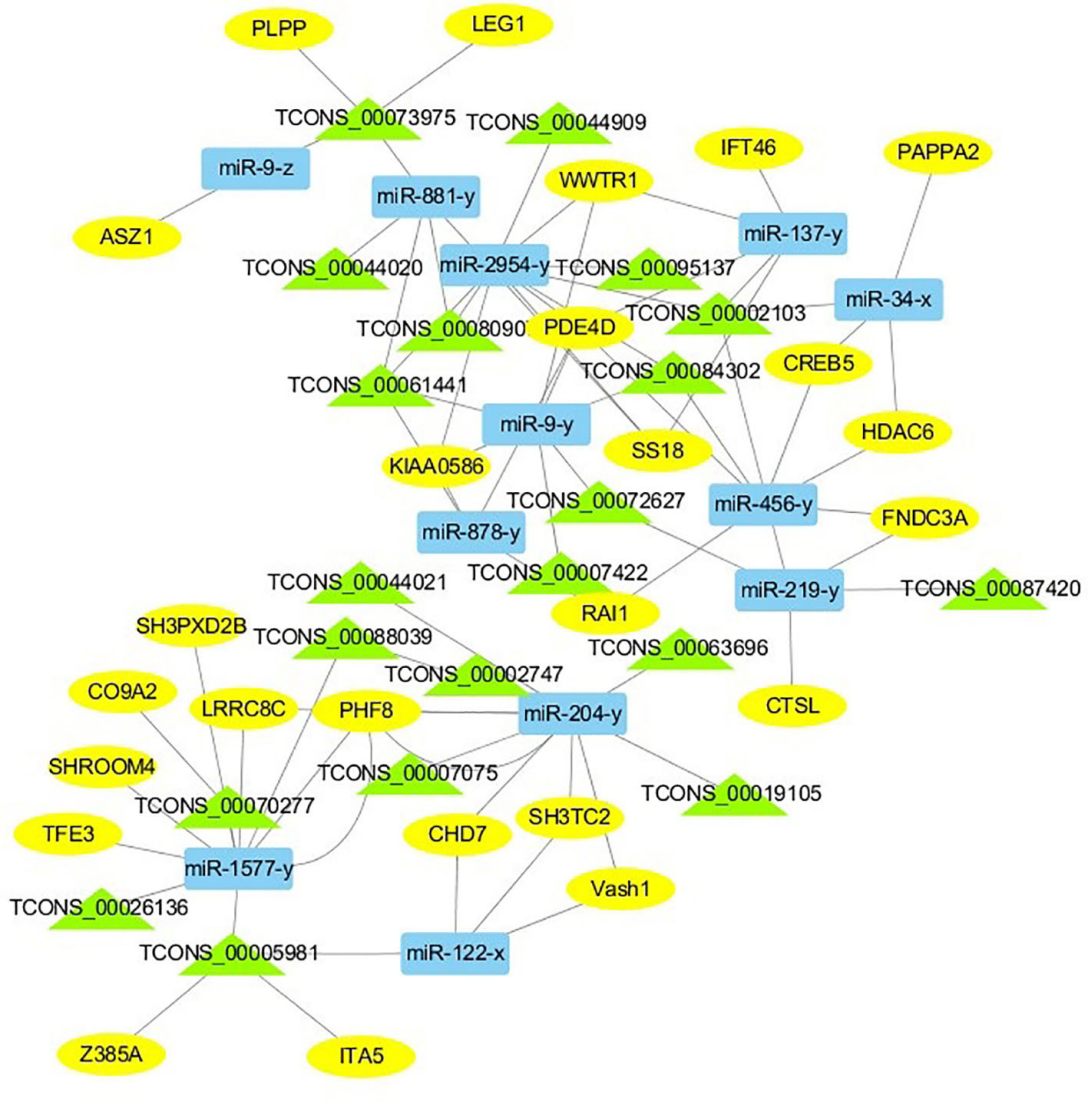
ceRNET of 60 d versus 90 d. Rectangles represent differentially expressed miRNAs, ellipses represent differentially expressed mRNAs, and the triangle represent differentially expressed lncRNAs. The gray lines represent the interactions between diverse RNAs.

**Figure 7:**
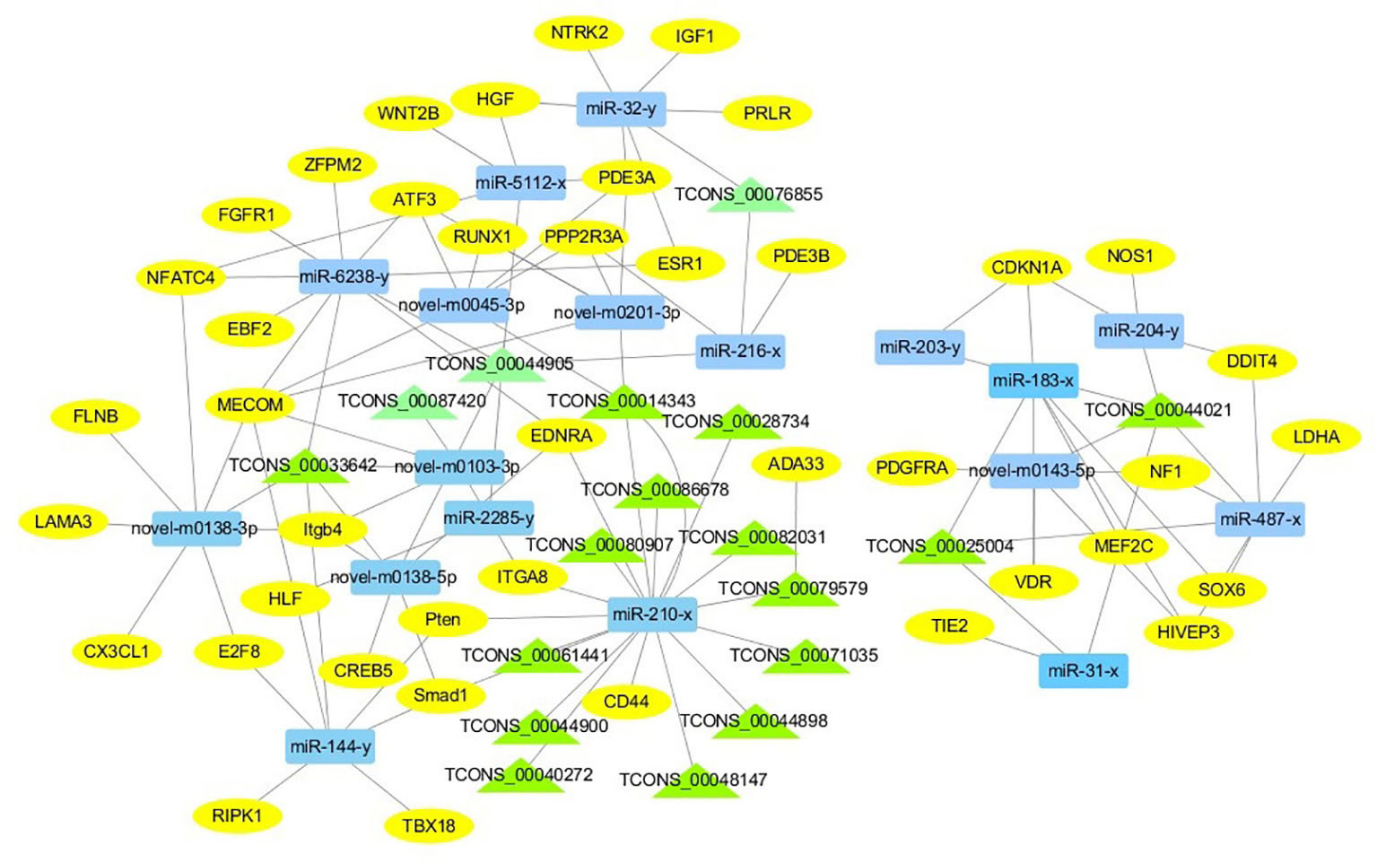
ceRNET of 30 d versus 90 d. Rectangles represent differentially expressed miRNAs, ellipses represent differentially expressed mRNAs, and the triangle represent differentially expressed lncRNAs. The gray lines represent the interactions between diverse RNAs.

For the construction of ceRNET related to antler development, a total of 182 miRNA-mRNA negative interaction pairs and 89 miRNA-lncRNA negative interaction pairs were screened for constructing ceRNET. In the 30 d versus 60 d, ceRNET consisting of 40 mRNAs, 17 miRNAs, and 9 lncRNAs was constructed (**Figure 5**). The number of genes enriched in the PI3K/AKT signaling pathway was the highest, followed by Hippo signaling pathway and cGMP/PKG signaling pathway. There were 8 DEGs (DDIT4, HGF, PRLR, PPP2R3A, CDKN1A, VEGFA, COL6A1, and Itgb4) significantly enriched in PI3K/AKT signaling pathway, some DEGs enriched in Hippo signaling pathway, cAMP signaling pathway, and PI3K/AKT signaling pathway. In the 60 d versus 90 d, the ceRNET included 25 mRNAs, 12 miRNAs, and 20 lncRNAs (**Figure 6**). Forty-two mRNAs, 18 miRNAs, and 18 lncRNAs were screened to constructed ceRNET for the 30 d versus 90 d (**Figure 7**). Among them, 9 DGEs (Pten, ITGA8, TIE2, NTRK2, IGF1, FGFR1, LAMA3, CREB5, and PDGFRA) were significantly enriched in PI3K/AKT signaling pathway, and 9 genes were enriched in MAPK signaling pathway.

## Discussion

CeRNET plays a widely regulatory role in tissue and organ development (Xu et al., 2016; Li et al., 2017; Liu et al., 2018; Miao et al., 2018). To interpret the interplay among diverse RNAs (mRNA, miRNA, and lncRNA) during the antler development, ceRNET was constructed for the first time. It provides a new perspective on the crosstalk among diverse RNAs. Due to the complexity of antler development and regeneration, there are many factors that affect the antler development and regeneration. Obviously, antler development requires the coordination between the protein-coding RNAs and non-coding RNAs. In our study, 17, 18 and 12 ceRNA pairs were screened to construct the ceRNET in the 30d versus 60d, 30d versus 90d, and 60d versus 90d, respectively. These protein-coding and non-coding RNAs regulate the antler development through mutual interaction and competition.

In the ceRNET, ceRNA pairs form a complex regulatory network by interacting with each other. Many lncRNAs and miRNAs co-target related genes that affect antler proliferation and development. The gene expression is regulated by more than one miRNA. Three diverse RNAs (miRNA, mRNA, and lncRNA) could communicate with each other by shared “MREs”, suggesting the possibility to answer questions related to antler development, and this mode of regulation has also been confirmed during antler regeneration (Hu et al., 2014b). An increasing evidence showed that lncRNAs exhibited tissue-specific expression (Derrien et al., 2012; Xu et al., 2016). Furthermore, our results revealed the lncRNA during the antler development from the perspective of temporal and special expression. 26, 21, 21 lncRNAs were specifically expressed during the 30 d, 60 d, and 90 d, respectively. Interestingly, same ceRNA pairs showed the significantly enhanced with each other in different periods, especially from the 30 d to 60 d, although the interact and competitive always existed.

Based on the DEGs we screened, ceRNA pairs related to antler development were obtained to construct ceRNET by GO enrichment analysis, and a large number of genes were mainly enriched in animal organ development, cellular developmental process, system development, and tissue development. Among them, the number of genes enriched in cellular development process was the largest. Moreover, the number of genes enriched in these four GO terms was greater in samples taken at times 30 d and 60 d during the antler development. While in the 90 d of antler development, the number of genes that exert developmental functions was significantly decreased. We also found that some genes not only played one role during the antler development, such as COL11A2, KCNMA1, TGFBR3, IHH, and GLI2, etc. The Col family genes are ubiquitously expressed in the five tissue layers of antler and three development periods (Price et al., 1996). However, the expression level of the same gene is different in three periods. The expression level of Collagen gene family was generally increased in the 60 d of antler compared to the 30 d. Among the DEGs screened, the col6a1, col18a1, and col11a2 that were enriched in the GO term of “cellular development” were significantly different in the 30 d versus 60 d, and COL11A2 has been shown to be highly correlated with the physiological and growth characteristics of antler (Hu et al., 2019). CDKN1, a regulator of collagen type X, was also identified (Lord et al., 2010), which shows the interaction between genes. Moreover, IHH may be involved in the rapid development of antler. IHH negatively regulates terminal chondrocyte differentiation through Pthrpr (Kobayashi et al., 2002). IHH regulates the differentiation of mesenchymal cells into precartilage in the process of antler development (Baojin et al., 2018).

Transcriptome analysis of three periods during the antler development can provide fundamental insights into development and regeneration. It is of great interest to study the genes that are enriched in the developmental process. In these expression profile of DEGs, some DEGs are significantly enriched in PI3K/AKT, cAMP, MAPK, cGMP/PKG, Wnt, and Hippo signaling pathways. Among them, most genes are enriched in PI3K/AKT signaling pathway. PI3K/AKT signaling pathway is involved in cell proliferation, growth, differentiation, and survival (Safdari et al., 2015). The previous study has shown that PI3K/AKT signaling pathway was most relevant to antler development (Li et al., 2012). Meanwhile, the ceRNET showed that the three comparison groups had the largest number of DEGs enriched in PI3K/AKT signaling pathway. Therefore, the identification of DEGs related to PI3K/AKT signaling pathway is of great significance for the study of candidate genes for the molecular mechanism of antler rapid development. It's worth noting that HGF, PRLR, Itgb4, and PPP2R3A are down-regulated from the 30 d to 60 d; on the contrary, DDIT4, CDKN1A, VEGFA, and COL6A1 were up-regulated from the 30 d to 60 d, which indicates that these genes are strongly related to antler development. Combined with the ceRNET, these interactions referred to 11 miRNA including miR-141, miR-144, miR-197, miR-200, miR-203, miR-4510, miR-457, and novel-m0138-5p .etc. There are 8 lncRNAs that interact with these 11 miRNAs, including TCONS_00017386, TCONS_00002747, TCONS_00084302, TCONS_00072627, TCONS_00076855, TCONS_00044905, TCONS_00001407, and TCONS_00026627. We reasonably speculate that these 8 genes effects the antler rapid development by interacting with 11 miRNAs and 8 lncRNAs. Moreover, MAPK signaling pathway has positive and negative effects on regulating cell proliferation and bone development, especially cartilage formation (Zhang et al., 2014). PRKCB and CDC25B were enriched in MAPK signaling pathway in the 30 d versus 60 d. Therefore, PRKCB and CDC25B may affect the antler development to some extent by affecting cartilage formation through interact with novel-m0138-5p, miR-144-x, miR-6238-y, TCONS_00076855, and TCONS_00044905. We surprisingly found that HGF, PRLR, Itgb4, PPP2R3A, DDIT4, and CDKN1A were also enriched in PI3K/AKT signaling pathway. Compared with other genes, CDKN1A and DDIT4 are highly expressed, which indicates that CDKN1A and DDIT4 play an important role in the whole antler development periods. In the ceRNET of 60 d versus 90 d, CREB5 was enriched in PI3K/AKT signaling pathway. Therefore, we hypothesize that CREB5 plays a major role in promoting antler development by interacting with miR-34-x, miR-456-x, TCONS_00002103, and TCONS_00084302. We also observed that transcription factors are involved in ceRNET in three periods, especially in the 30 d versus 90 d during the growth and development of antler, such as SOX6, HIVEP3, RUNX1, MECOM, and VD. It is reasonable to speculate that transcription factor's regulatory potency is much larger than anticipated and these transcription factors also play an indispensable role in the growth and development of antler. However, how transcription factors play a role in antler development has not been experimentally verified.

CeRNET related to antler development was constructed in this study. During the antler development, ceRNET activated the expression of protein-coding RNAs; on the other hand, the regulatory effects of non-coding RNAs were used to control the development of cells towards non-cancerous, promoting the rapid development of antler. Due to the complexity of gene interactions, changes in the expression levels of either non-coding RNAs or coding RNAs may lead to the disorder of antler development. Furthermore, the construction of ceRNET will enable us to better understand the interaction between the diverse RNAs, which provides a further basis for revealing the regulatory mechanism of antler development. It may provide a new perspective for the diagnosis and treatment of human tumor diseases.

## Data Availability Statement

The raw data has been submitted to the National Center for Biotechnology Information (NCBI) Sequence Read Archive (SRA), and the accession number is PRJNA552211 and PRJNA552158.

## Ethics Statement

The animal study was reviewed and approved by Northeast Forestry University Ethics Committee. Written informed consent was obtained from the owners for the participation of their animals in this study.

## Author Contributions

HL and RH conceived and designed this experiment. HL, RH, LH, and SW collected the antler samples from sika deer. RH and LH performed experiments. The manuscript was prepared by RH. All authors read and approved the final manuscript.

## Funding

This study was supported by the Natural Science Foundation of Heilongjiang Province (C2017012). The funders had no role in the design of the study and collection, analysis, decision to publish, interpretation of data or preparation of the manuscript.

## Conflict of Interest

The authors declare that the research was conducted in the absence of any commercial or financial relationships that could be construed as a potential conflict of interest.

## Abbreviations

ceRNET, CeRNA regulatory network; DEG, differentially expressed genes; DE miRNAs, differentially expressed miRNAs; lncRNAs, differentially expressed lncRNAs; lncRNA, long non-coding RNAs; MREs, microRNA recognition elements; (PCC), Pearson correlation coefficient.
