# Supplementary material for: Whole Transcriptome Analysis of Mesenchyme Tissue in Sika Deer Antler Revealed the CeRNAs Regulatory Network Associated With Antler Development
Source: Front Genet. 2020 Feb 18;10:1403. doi: 10.3389/fgene.2019.01403 (PMC7040488; doi:10.3389/fgene.2019.01403)

TableS1 Primer pairs of differentially expressed genes used for qRT-PCR validation

| Name | Primer 5’→3’ |
| --- | --- |
| GAPDH F | AGATGGTGAAGGTCGGAGTG |
| GAPDH R | CCTTTCCATTGATGACGAGC |
| KCNMA1 F | TGAGATCTGCTTGCTGTGTGA |
| KCNMA1 R | ATGATCAATGATTGCCCACTCG |
| Smad1 F | GGATTCGATTGCCTCACTGC |
| Smad1 R | ATGCCGAGGTCCTGAAAAGT |
| FGFR F | GAGAGGAGGTTGGCTGTGAA |
| FGFR R | TGCCTTCTTTCCCTGCCTTT |
| ss18 F | GTTACAACCATTCCGTGCCG |
| ss18 R | TGTTGAGAAGGAGGCTGCTG |
| CREB5 F | TGCTTCAGTTCTGCCAGATT |
| CREB5 R | ATTCCAGGCCAAGCGATGAT |
| VEGFA F | GAGAGAGTCTGGCATCGTCTT |
| VEGFA R | GTCAGCTGTCTTTCTGTCCG |
| U6 F | GGAACGATACAGAGAAGATTAGC |
| U6 R | TGGAACGCTTCACGAATTTGCG |
| miR-141-y | TAACACTGTCTGGTAAAGATGG |
| miR-137-y | TTATTGCTGGAGAATACGCGTAG |
| miR-881-y | TAACTGTGGCATTTCTGAATAGA |
| novel-m0143-5p | CAGGAGCCTCGGCTGACTGCT |
| miR-4510-x | TGAGGGAGTAGGTTGTATGGTT |
| miR-210-x | AGCCACTGCCCACCGCACACTG |
| TCONS_00001407 F | ATAGGAGCTGAAGAGTCCGC |
| TCONS_00001407 R | ATCCCCTGGAACTTAGGCATC |
| TCONS_00072627 F | GTTCCCACTTTCGGTTTTGGG |
| TCONS_00072627 R | AAGCACAGTATTGGGGTGGG |
| TCONS_00033642 F | TGACCGGCACTAGCTTTAGG |
| TCONS_00033642 R | TGGTGCCACGGTTTATCCAA |
| TCONS_00014343 F | CCATTTCCCGGTTTGGGAGA |
| TCONS_00014343 R | AGCCTTCCCAAACTGTACCC |
| TCONS_00002103 F | TGCGCCCGCAAAAATGATAG |
| TCONS_00002103 R | GCTGACAGTCCCTTGATCGT |
| TCONS_00044909 F | ACGCTCTGTAACCTCACACC |
| TCONS_00044909 R | TGCAAGCAACACTCAGCAAC |

TableS2 Overview of RNA-seq results

| sample | Clean data | Q30(%) | Mapping |
| --- | --- | --- | --- |
| 30d-1 | 14247965635 | 13760955591 (96.58%) | 73.03% |
| 30d -2 | 12871321538 | 12396363042 (96.31%) | 71.17% |
| 30d -3 | 12571250466 | 12135443647 (96.53%) | 72.40% |
| 60d-1 | 15577077840 | 14992548220 (96.25%) | 71.27% |
| 60d -2 | 13881852748 | 13393392924 (96.48%) | 71.85% |
| 60d -3 | 12578243734 | 12124406466 (96.39%) | 72.97% |
| 90d-1 | 13103249014 | 12653311383 (96.57%) | 72.80% |
| 90d -2 | 14563031108 | 14070126339 (96.62%) | 72.27% |
| 90d -3 | 14116163832 | 13618931886 (96.48%) | 72.73% |

TableS3 Overview of small RNA-seq results

| Sample | Known miRNA | Ratio to the reference genome (%) |
| --- | --- | --- |
| 30d-1 | 7739957 (71.36%) | 71.87 |
| 30d -2 | 9916601 (84.01%) | 77.57 |
| 30d -3 | 9023114 (80.71%) | 77.23 |
| 60d-1 | 6267260 (62.53%) | 68.38 |
| 60d -2 | 7060446 (63.45%) | 67.77 |
| 60d -3 | 5683247 (72.76%) | 72.78 |
| 90d-1 | 6959318 (66.90%) | 70.43 |
| 90d -2 | 7812489 (70.54%) | 71.75 |
| 90d -3 | 7488269 (72.24%) | 72.01 |

FigureS1 Validation of differentially expressed mRNAs, miRNAs, lncRNAs by qRT-PCR


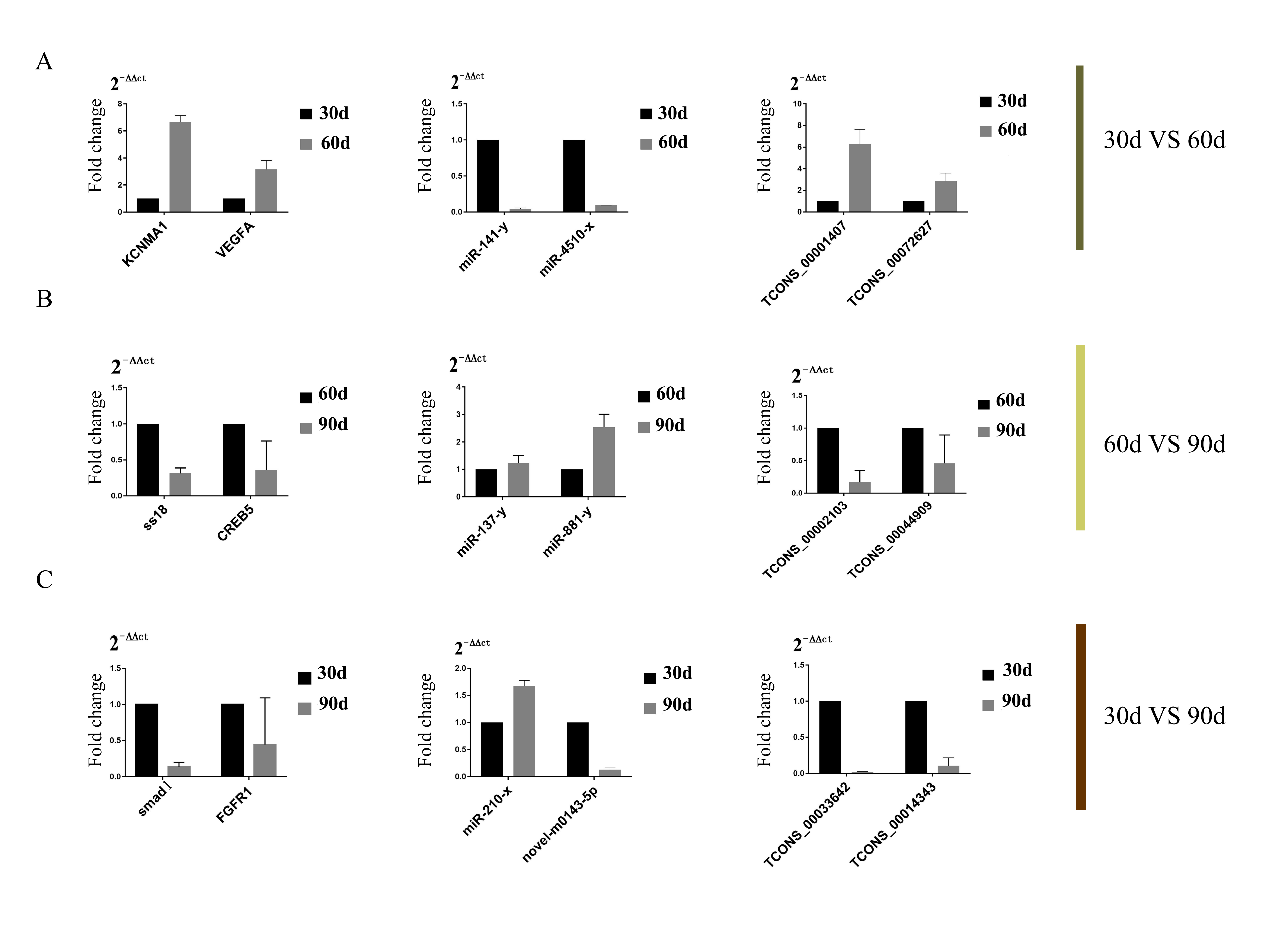

Supplement: Supplementary file 1 [file DataSheet_1.docx]
